# Supplementary material for: Cross-Cultural Differences between Italian and UK Consumer Preferences for ‘Big Top’ Nectarines in Relation to Cold Storage
Source: Foods. 2022 Aug 12;11(16):2424. doi: 10.3390/foods11162424 (PMC9407176; doi:10.3390/foods11162424)
Supplement: Supplementary file 1 [file foods-11-02424-s001.zip › foods-1833881-supplementary.pdf]

Article

Cross-Cultural Differences between Italian and UK Consumer Preferences for 'Big Top' Nectarines in Relation to Cold Storage

Sarah R. Christofides [1], Anita Setarehnejad [2], Ruth Fairchild [2], Innocenzo Muzzalupo [3], Leonardo Bruno [4], Antonella Muto [4], Adriana Chiappetta [4], Maria B. Bitonti [4], Carsten T. Müller [1], Hilary J. Rogers [1,*] and Natasha D. Spadafora [4,5]

[1] School of Biosciences, Cardiff University, Sir Martin Evans Building, Museum Avenue, Cardiff, CF10 3AX, UK
[2] Department of Healthcare and Food, School of Health & Sport Sciences, Cardiff Metropolitan University, Cardiff, CF5 2YB, UK
[3] Centro di Ricerca Foreste e Legno, Consiglio per la Ricerca in Agricoltura e L'analisi Dell'economia Agraria, 87036 Rende, Italy
[4] Department of Ecology, University of Calabria, 87036 Rende, Italy
[5] Department of Chemical, Pharmaceutical and Agricultural Sciences, University of Ferrara, 44121 Ferrara, Italy
* Correspondence: rogershj@cf.ac.uk

Citation: Christofides, S.R.; Setarehnejad, A.; Fairchild, R.; Muzzalupo, I.; Bruno, L.; Muto, A.; Chiappetta, A.; Bitonti, M.B.; Müller, C.T.; Rogers, H.J.; et al. Cross-Cultural Differences between Italian and UK Consumer Preferences for 'Big Top' Nectarines in Relation to Cold Storage. *Foods* **2022**, *11*, 2424. https://doi.org/10.3390/foods11162424

Academic Editor: Wendy Wismer

Received: 10 July 2022
Accepted: 6 August 2022
Published: 12 August 2022

Publisher's Note: MDPI stays neutral with regard to jurisdictional claims in published maps and institutional affiliations.

Copyright: © 2022 by the authors. Submitted for possible open access publication under the terms and conditions of the Creative Commons Attribution (CC BY) license (https://creativecommons.org/licenses/by/4.0/).

Supplmentary Data S1A. Questionnaire used at UK sites

Prior to running the taste panel interested participants will be asked:

- Do you eat stone fruit (peaches)? If yes, continue. If no, thank them.
- Have you had any past allergic reactions to fruit? If no, continue. If yes, no thank you.
- Would you like to taste some ripe stone fruit for a university research project? If yes, continue and give the form below. If no, thank them.

Ask consumer to read information sheet and sign consent form.

CONSUMER TEST of PEACHES

Ethics Protocol number **CSHS/9492 for preference testing**
Ethics application number **Sta-1353/SREC 1906-02** ....

This taste panel will ask your opinion (like/dislike) of three different samples of peach and what you like or dislike about each. So that we can classify your answers we will also ask you a few questions about yourself and your fruit buying/consumption habits.

All of the answers you give are anonymous and cannot be traced back to you when you return your questionnaire. The anonymous data we collect will be retained for further analysis.

If you return this questionnaire to us then we presume you are consenting to your anonymous answers being used in publications, presentations and teaching, from this point forward (e.g. we do not intend to destroy the data).

If you **do not wish your data to be included in this study then please do not return the questionnaire to us.**

Please circle the answer that best represents you or indicate if other:

Q1. Gender Male Female

Q2. Age Range (years)
18-29
30-39

40-49
50-59
60 or older

Q3. Choose one option that best describes your ethnic group or background

……. a Caucasian
……. b Asian
……. c Hispanic
……. d Black
……. e Native American
……. f Other (please describe) ………………………………………..

Q4. How would you describe your nationality? ………………………………………

Q5. How often do you eat peaches in the UK in season e.g. summer?

More than once per week

Once per week

1-3 times per week

Less than once per month

Q6. When selecting peaches do you look for specific named varieties? Yes /No

If yes, state which varieties.............

Q7. When selecting peaches what are the most important factors for your choice? Please choose as many as relevant.

Ripeness/ Colour/ Size/ Shape/ Price / Aroma/ Texture when picked up/ Taste/other

Q8. Please taste the samples in the order stated below and indicate your overall liking for each sample
using the chart provided, where **1 equals extremely disliked and 9 equals extremely liked**.

Sample	Extremely Disliked								Extremely liked
xxx	1	2	3	4	5	6	7	8	9
xxx	1	2	3	4	5	6	7	8	9
xxx	1	2	3	4	5	6	7	8	9

Q9. For each sample circle all the descriptors that best describe each peach

Varieties	*Choose the most appropriate descriptors by circling all that apply*
xxx	Peachy aroma, Sweet, Acid, Bitter, Astringent, Juicy, Crunchy, Chewy/Fibrous skin, Firm flesh, Soft flesh
xxx	Peachy aroma, Sweet, Acid, Bitter, Astringent, Juicy, Crunchy, Chewy/Fibrous skin, Firm flesh, Soft flesh
xxx	Peachy aroma, Sweet, Acid, Bitter, Astringent, Juicy, Crunchy, Chewy/Fibrous skin, Firm flesh, Soft flesh

Many thanks for your time.

If you are happy for your data to be included in the study please return this questionnaire to the researchers.

If you have further questions about the study please contact:

Dr Hilary Rogers rogershj@cf.ac.uk

Supplementary Data S1B. Questionnaire used at IT site

CONSUMER TEST

Consumatore N. ………. *Luogo* …………………………*Data* … / … / ……

Chiedere se il consumatore mangia frutti di drupacee (pesche e/o nettarine).
Se sì, continua. Se no, grazie
Chiedi se il consumatore desidera assaggiare qualche frutto maturo per un progetto di ricerca Universitario.
Se sì, continua. Se no, grazie
Fai domande demografiche

D.1 Genere

……. ***a*** Maschio

……. ***b*** Femmina

D2. Razza

……. ***a*** Caucasica ……………………………. Nazionalità

……. ***b*** Asiatica ……………………………. Nazionalità

……. ***c*** Spagnola ……………………………. Nazionalità

……. ***d*** Nera ……………………………. Nazionalità

……. ***e*** Nativo americano ……………………………. Nazionalità

……. ***f*** altro ……………………………. Nazionalità

D3. Fascia d'età

……. ***a*** Sotto 19

……. ***b*** 20-29

……. ***c*** 30-39

……. ***d*** 40-49

……. ***e*** 50-59

……. ***f*** 60 o più

D4. Professione

……. ***a*** Studente

……. ***b*** Impiegato

D5. Con quale frequenza mangi pesche di stagione in Italia, ad es. estate?

……. ***a*** Più di una volta a settimana
……. ***b*** Una volta a settimana
……. ***c*** 2-3 volte a settimana
……. ***d*** Meno di una volta al mese

D6. Preferenza di una varietà specifica?

……. ***a*** Si
……. ***b*** No

D7. Quando selezioni le pesche quali sono i fattori implicanti nella scelta delle pesche?
(Si prega di scegliere come tanti quanto rilevanti).

……..***a*** Gusto
……. ***b*** Maturazione
……. ***e*** Colore
……. ***d*** Dimensione
……. ***e*** Forma
……. ***f*** Prezzo
……. ***g*** Aroma
……. ***h*** Consistenza
……. ***i*** Altro

I campioni devono essere preparati a parte lontano dalla vista del consumatore e serviti in bicchieri di plastica con coperchio (un pezzo per campione).

Dì al consumatore che assaggerà quattro campioni di frutta matura. Per ogni campione chiedi se lui / lei "Mi piace", "Non mi piace" o "Mi è indifferente".

Chiedigli il grado di gradimento / sgradimento: *leggermente*, *moderatamente*, *molto* o *estremamente*. Fagli sorseggiare dell'acqua in bottiglia.

Ripeti la prova con il secondo, terzo, quarto, … campione (appena raccolto, X day a 1C e 4°C)

Consumatore N. ………. *Luogo ………………………….Data … / … / ……*

**Varietà**	**Estremamente sgradevole**	**Molto sgradevole**	**Moderatamente sgradevole**	**Appena sgradevole**	**Indifferente**	**Appena gradevole**	**Moderatamente gradevole**	**Molto gradevole**	**Estremamente gradevole**
	**1**	**2**	**3**	**4**	**5**	**6**	**7**	**8**	**9**
Big Top 1									
Big Top 2									
Big Top 3									

**Varietà**	**Scegli i descrittori più adeguati:**
Big Top 1	Dolce, Acida, Astringente, Succosa, Croccante, Consistente, Dura, Tenera, Fragrante, Aromatica, Amara, Fibrosa
Big Top 2	Dolce, Acida, Astringente, Succosa, Croccante, Consistente, Dura, Tenera, Fragrante, Aromatica, Amara, Fibrosa

Big Top 3	Dolce, Acida, Astringente, Succosa, Croccante, Consistente, Dura, Tenera, Fragrante, Aromatica, Amara, Fibrosa

Table S1. Participant information

		Site				
	information	CF 1	CF 2	IT	Tot	%
	Participants	103	101	155	359	100
Gender	Male	39	48	87	174	48.47
	Female	58	53	68	179	49.86
	non disclosed gender	6	0	0	6	1.67
Age	18–29 years	19	33	113	165	45.96
	30–39 years	30	19	12	61	16.99
	40–49 years	21	25	10	56	15.60
	50–59 years	24	20	15	59	16.43
	60+ years	9	4	5	18	5.01
Ethnicity	Caucasian	91	89	122	302	84.12
	Asian	5	3	8	16	4.46
	Hispanic	0	2	11	13	3.62
	Black	2	1	14	17	4.74
	Other	3	4	0	7	1.95
	NA	2	2	0	4	1.11
Nationality	British	87	69		156	43.45
	Italian		3	113	116	32.31
	Other	16	29	42	87	24.23

Table S2. Results of univariate tests on whether the frequency of purchase and nation (Italy vs UK) affects the importance of each characteristic in influencing the decision to purchase.

	**Aroma**		**Best before**		**Blemishes**		**Colour**		**Other**		**Price**		**Retailer**	
	LR	*P*	LR	*P*	LR	*P*	LR	*P*	LR	*P*	LR	*P*	LR	*P*
Gender	1.05	>0.999	0.38	>0.999	1.99	0.92	0.4	>0.999	1.2	0.992	3.29	0.992	4.3	0.4
Country	**13.6**	**0.001**	**35.1**	**0.001**	**18**	**0.01**	0.1	0.929	5.8	0.159	**20.4**	**0.001**	2.4	0.8
Frequency	10.8	0.078	3.9	0.834	3.35	0.89	1.4	0.991	0.9	0.998	1.11	0.998	0.6	1
Country: Frequency	0.59	0.998	1.4	0.993	1.09	0.99	0.3	0.998	0	0.998	2.89	0.948	2	1

	**Ripeness**		**Shape**		**Size**		**Taste**		**Texture**		**Variety**	
	LR	*P*	LR	*P*	LR	*P*	LR	*P*	LR	*P*	LR	*P*
Gender	10.6	0.213	0.48	>0.999	0.27	>0.999	1.4	>0.999	0.3	>0.999	2.99	0.999
Country	**32.6**	**0.001**	0.35	0.929	16.3	0.08	**53**	**0.001**	6.5	0.306	**56**	**0.001**
Frequency	5.08	0.63	0.56	0.998	3.36	0.89	0.3	0.998	0.6	0.998	7.09	0.335
Country: Frequency	0.17	0.998	6.17	0.444	0.15	1	1.7	0.99	2.3	0.984	6.24	0.444

Comparisons are based on likelihood ratio tests. *P* values are adjusted using a step-down resampling procedure.

Table S3. Top candidate models (ΔAICc <2) used in model averaging to predict peach ratings based on peach characteristics.

Rank	Terms	DF	log*L*	AICc	*Δ*AICc	Weight
1	Acid + Aroma + Astringent + Bitter + Crunchy + Juicy + Sweet	17	−1566.99	3168.67	0	0.26
2	Acid + Aroma + Astringent + Bitter + Crunchy + Fibrous + Juicy + Sweet	18	−1566	3168.77	0.1	0.25
3	Acid + Aroma + Astringent + Bitter + Crunchy + Juicy + Soft + Sweet	18	−1566.63	3170.02	1.35	0.13
4	Aroma + Astringent + Bitter + Crunchy + Juicy + Sweet	16	−1568.73	3170.06	1.39	0.13
5	Acid + Aroma + Astringent + Bitter + Crunchy + Fibrous + Juicy + Soft + Sweet	19	−1565.61	3170.07	1.4	0.13
6	Aroma + Astringent + Bitter + Crunchy + Fibrous + Juicy + Sweet	17	−1567.93	3170.55	1.89	0.1

DF = degrees of freedom, Log*L* = log-likelihood, AICc = Akaike's information criterion with the small sample bias adjustment, *Δ*AICc = difference in AICc between the current model and the best model, Weight = Akaike weight.

Table S4. Co-occurrence of peach characteristics.

Comparison	*Both*	*Char1*	*Char2*	*Neither*	*OddsRatio*	*P*	*P.adj*
**Aroma vs Sweet**	**171**	**97**	**343**	**466**	**2.39**	**<0.001**	**<0.001**
**Aroma vs Acid**	**19**	**249**	**110**	**699**	**0.49**	**0.005**	**0.010**
**Aroma vs Bitter**	**8**	**260**	**81**	**728**	**0.28**	**<0.001**	**<0.001**
**Aroma vs Astringent**	**6**	**262**	**51**	**758**	**0.34**	**0.007**	**0.015**
**Aroma vs Juicy**	**95**	**173**	**161**	**648**	**2.21**	**<0.001**	**<0.001**
Aroma vs Crunchy	148	120	435	374	1.06	0.724	0.740
Aroma vs Fibrous	38	230	80	729	1.50	0.055	0.083
Aroma vs Firm	133	135	416	393	0.93	0.622	0.683
**Aroma vs Soft**	**53**	**215**	**84**	**725**	**2.13**	**<0.001**	**<0.001**
**Sweet vs Acid**	**21**	**493**	**108**	**455**	**0.18**	**<0.001**	**<0.001**
**Sweet vs Bitter**	**10**	**504**	**79**	**484**	**0.12**	**<0.001**	**<0.001**
**Sweet vs Astringent**	**12**	**502**	**45**	**518**	**0.28**	**<0.001**	**<0.001**
**Sweet vs Juicy**	**185**	**329**	**71**	**492**	**3.89**	**<0.001**	**<0.001**
**Sweet vs Crunchy**	**250**	**264**	**333**	**230**	**0.65**	**0.001**	**0.001**
Sweet vs Fibrous	50	464	68	495	0.78	0.241	0.312
**Sweet vs Firm**	**228**	**286**	**321**	**242**	**0.60**	**<0.001**	**<0.001**
**Sweet vs Soft**	**108**	**406**	**29**	**534**	**4.89**	**<0.001**	**<0.001**
**Acid vs Bitter**	**18**	**111**	**71**	**877**	**2.00**	**0.017**	**0.029**
Acid vs Astringent	8	121	49	899	1.21	0.673	0.722
**Acid vs Juicy**	**14**	**115**	**242**	**706**	**0.36**	**<0.001**	**<0.001**
Acid vs Crunchy	73	56	510	438	1.12	0.573	0.645
Acid vs Fibrous	10	119	108	840	0.65	0.291	0.364
Acid vs Firm	68	61	481	467	1.08	0.708	0.740
**Acid vs Soft**	**4**	**125**	**133**	**815**	**0.20**	**<0.001**	**<0.001**
Bitter vs Astringent	7	82	50	938	1.60	0.317	0.385
**Bitter vs Juicy**	**11**	**78**	**245**	**743**	**0.43**	**0.009**	**0.017**
Bitter vs Crunchy	54	35	529	459	1.34	0.222	0.303
Bitter vs Fibrous	16	73	102	886	1.90	0.033	0.053
Bitter vs Firm	53	36	496	492	1.46	0.097	0.142
**Bitter vs Soft**	**4**	**85**	**133**	**855**	**0.30**	**0.012**	**0.023**
**Astringent vs Juicy**	**4**	**53**	**252**	**768**	**0.23**	**0.001**	**0.003**
**Astringent vs Crunchy**	**26**	**31**	**557**	**463**	**0.70**	**0.219**	**0.303**
Astringent vs Fibrous	6	51	112	908	0.95	>0.999	>0.999
Astringent vs Firm	32	25	517	503	1.25	0.496	0.573
Astringent vs Soft	2	55	135	885	0.24	0.038	0.059
**Juicy vs Crunchy**	**94**	**162**	**489**	**332**	**0.39**	**<0.001**	**<0.001**

**Juicy vs Fibrous**	**18**	**238**	**100**	**721**	**0.55**	**0.022**	**0.036**
**Juicy vs Firm**	**75**	**181**	**474**	**347**	**0.30**	**<0.001**	**<0.001**
**Juicy vs Soft**	**87**	**169**	**50**	**771**	**7.92**	**<0.001**	**<0.001**
**Crunchy vs Fibrous**	**51**	**532**	**67**	**427**	**0.61**	**0.014**	**0.025**
**Crunchy vs Firm**	**363**	**220**	**186**	**308**	**2.73**	**<0.001**	**<0.001**
**Crunchy vs Soft**	**21**	**562**	**116**	**378**	**0.12**	**<0.001**	**<0.001**
Fibrous vs Firm	54	64	495	464	0.79	0.243	0.312
Fibrous vs Soft	18	100	119	840	1.27	0.380	0.450
**Firm vs Soft**	**13**	**536**	**124**	**404**	**0.08**	**<0.001**	**<0.001**

Both = number of responses which were 'yes' for both characters; Char1 = number of responses which were 'yes' only for the characteristic on the left hand side; Char2 = number of responses which were 'yes' only for the character on the right hand side; Neither = number of responses which were 'no' for both characters. Fisher's exact tests are presented for each pair; adjusted P-values were corrected by the Benjamini–Hochberg method. Significant pairs are indicated in bold.

Table S5. Univariate test results from the *manyglm* model regressing site and treatment against peach characteristics.

	**Acid**		**Aroma**		**Astringent**		**Bitter**		**Crunchy**	
	LR	*P*	LR	*P*	LR	*P*	LR	*P*	LR	*P*
Site	3.78	0.38	**91.71**	**0**	1.13	0.8	0.11	0.96	**131.6**	**0**
Treatment	3.41	0.2	**25.91**	**0**	**9.36**	**0.04**	**19.6**	**0**	**18.86**	**0**
Site: Treatment	**15.7**	**0.03**	**21.4**	**0.01**	9.33	0.21	8.03	0.22	**26.84**	**0**

	**Fibrous**		**Firm**		**Juicy**		**Soft**		**Sweet**	
	LR	*P*	LR	*P*	LR	*P*	LR	*P*	LR	*P*
Site	**21.6**	**0**	**103.5**	**0**	**24.91**	**0**	**92.3**	**0**	**16.56**	**0.01**
Treatment	5.21	0.15	**8.9**	**0.04**	**20.83**	**0**	**34.4**	**0**	**40.96**	**0**
Site: Treatment	3.55	0.52	**31.06**	**0**	**31.27**	**0**	11.6	0.13	**25.95**	**0**

Comparisons are based on likelihood ratio tests. *P*-values are adjusted for multiple testing via a step-down resampling procedure.

Table S6. Difference in how each character affected sensorial rating, in relation to consumer characteristics.

Character	Predictor	LR Chisq	D.F.	*Pr*(>Chisq)	*P*.adj
Aroma	Aroma	0.33	1	0.566	0.885
Aroma	Country	0.387	1	0.534	0.885
Aroma	Frequency	2.559	4	0.634	0.937
Aroma	Gender	0.001	1	0.973	0.985
Aroma	Aroma:Country	NA	0	NA	NA
Aroma	Aroma:Frequency	0.23	3	0.973	0.985
Sweet	Taste	1.593	1	0.207	0.655
Sweet	Ripeness	5.731	1	0.017	0.468
Sweet	Country	0	1	0.985	0.985
Sweet	Frequency	0.4	4	0.982	0.985
Sweet	Gender	1.941	1	0.164	0.655
Sweet	Taste:Country	1.041	1	0.308	0.747
Sweet	Taste:Frequency	1.454	3	0.693	0.971
Sweet	Ripeness:Country	2.25	1	0.134	0.655
Sweet	Ripeness:Frequency	10.451	3	0.015	0.468
Acid	Taste	0.135	1	0.714	0.971
Acid	Country	0.044	1	0.833	0.985
Acid	Frequency	3.053	3	0.384	0.869
Acid	Gender	2.047	1	0.152	0.655
Acid	Taste:Country	2.25	1	0.134	0.655
Acid	Taste:Frequency	2.699	3	0.44	0.885
Bitter	Taste	4.014	1	0.045	0.538
Bitter	Country	1.288	1	0.256	0.697
Bitter	Frequency	9.611	4	0.048	0.538
Bitter	Gender	1.559	1	0.212	0.655
Bitter	Taste:Country	5.356	1	0.021	0.468
Bitter	Taste:Frequency	0.31	3	0.958	0.985
Astringent	Taste	2.611	1	0.106	0.655
Astringent	Country	1.494	1	0.222	0.655
Astringent	Frequency	2.128	3	0.546	0.885
Astringent	Gender	0.023	1	0.878	0.985
Astringent	Taste:Country	0.166	1	0.684	0.971
Astringent	Taste:Frequency	4.349	2	0.114	0.655
Juicy	Texture	0.01	1	0.92	0.985
Juicy	Ripeness	1.8	1	0.18	0.655
Juicy	Country	1.636	1	0.201	0.655
Juicy	Frequency	3.923	4	0.416	0.885
Juicy	Gender	4.028	1	0.045	0.538

Juicy	Texture:Country	1.569	1	0.21	0.655
Juicy	Texture:Frequency	4.56	4	0.335	0.787
Juicy	Ripeness:Country	0.067	1	0.795	0.985
Juicy	Ripeness:Frequency	2.63	3	0.452	0.885
Crunchy	Texture	0.007	1	0.931	0.985
Crunchy	Country	3.012	1	0.083	0.655
Crunchy	Frequency	3.182	4	0.528	0.885
Crunchy	Gender	0.312	1	0.576	0.885
Crunchy	Texture:Country	0.095	1	0.758	0.985
Crunchy	Texture:Frequency	1.639	4	0.802	0.985
Fibrous	Texture	0.457	1	0.499	0.885
Fibrous	Country	0.4	1	0.527	0.885
Fibrous	Frequency	0.999	4	0.91	0.985
Fibrous	Gender	0.141	1	0.707	0.971
Fibrous	Texture:Country	1.434	1	0.231	0.655
Fibrous	Texture:Frequency	2.025	3	0.567	0.885
Firm	Texture	0.003	1	0.957	0.985
Firm	Country	1.067	1	0.302	0.747
Firm	Frequency	2.858	4	0.582	0.885
Firm	Gender	0.037	1	0.847	0.985
Firm	Texture:Country	2.255	1	0.133	0.655
Firm	Texture:Frequency	3.674	4	0.452	0.885
Soft	Texture	0	1	0.984	0.985
Soft	Ripeness	2.09	1	0.148	0.655
Soft	Country	1.63	1	0.202	0.655
Soft	Frequency	3.189	4	0.527	0.885
Soft	Gender	0.004	1	0.952	0.985
Soft	Texture:Country	1.7	1	0.192	0.655
Soft	Texture:Frequency	1.328	4	0.857	0.985
Soft	Ripeness:Country	1.226	1	0.268	0.702
Soft	Ripeness:Frequency	1.936	3	0.586	0.885

Each sensorial character was regressed against respondents' country, gender, frequency of purchase and which characteristics they named as important in purchase decisions. For each sensorial characteristic, only relevant purchase characteristics were included in the model, e.g. texture and ripeness were included in the model for softness whereas aroma was not.

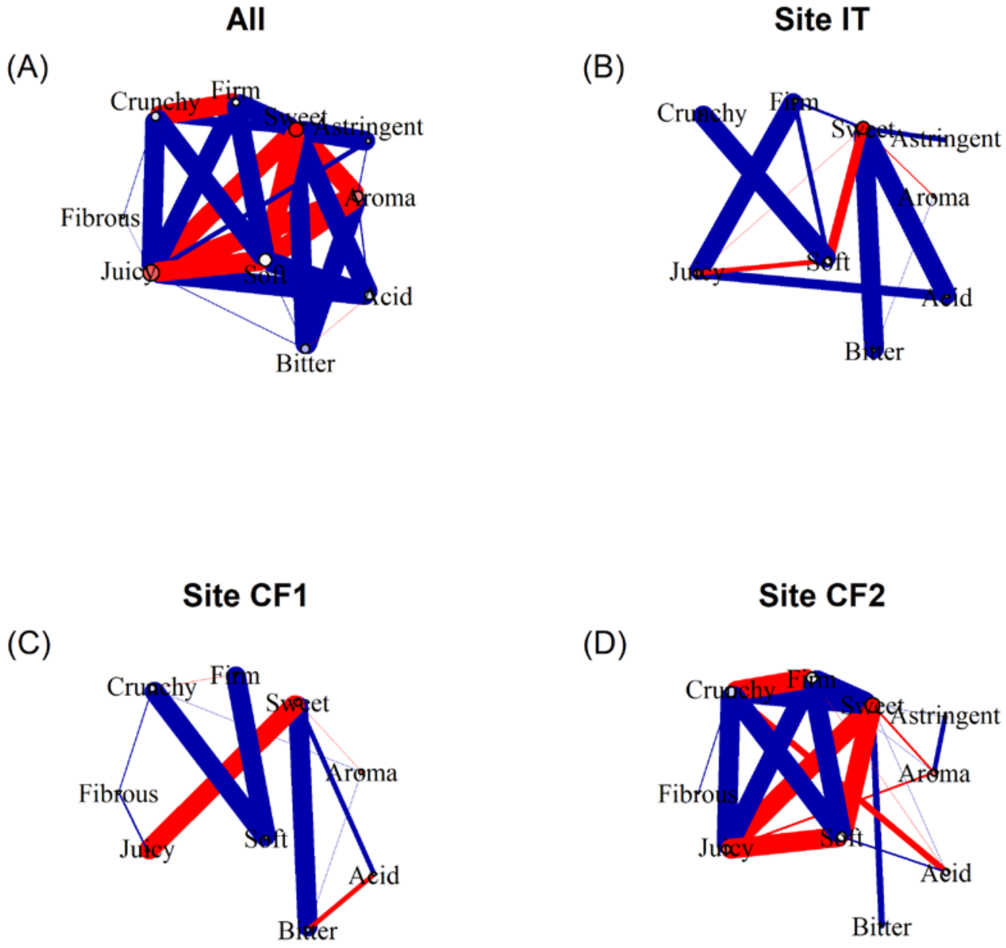

Figure S1. Co-occurrence frequency of peach characteristics by site. (A) all sites together, (B) site IT, (C) site CF1, (D) site CF2. Only significant associations are shown; for a full list see Table S4. Edge widths are inversely proportional to *P* values (thick edges indicate low *P* values). Red edges indicate that a pair of nodes co-occurred more often than expected by chance; blue edges indicate they co-occurred less often than expected by chance. Node colour for each characteristic is proportional to the corresponding parameter value in Figure 5: blue indicates a negative effect on hedonic rating and red a positive effect, while the strength of colour corresponds to the strength of the effect.
